# A Model Analysis of Tensile Stress in the Toadfish Vestibular Membranes

**DOI:** 10.1155/2011/519293

**Published:** 2011-06-08

**Authors:** Daniel J. Pender

**Affiliations:** The Department of Otolaryngology-Head and Neck Surgery, Columbia University Medical Center, New York, NY 10032, USA

## Abstract

*Background*. A theoretical model analysis of stress in the vestibular membranes has identified a geometrical stress factor incorporating shape, size, and thickness that can be used to assess peak stress in the various chambers. *Methods*. Using published measurements of the toadfish vestibular membranes made during surgery, the geometrical stress factor can be evaluated for each chamber based on the model. *Results*. The mean geometrical stress factor is calculated to be the lowest in the semicircular canal (4.4), intermediate in the ampulla (6.0), and the highest in the utricle (17.4). *Conclusions*. The model predicts that substantial hoop stress disparities exist in the toadfish vestibular labyrinth. Stress is least in the semicircular canal, which therefore appears to be the structure with greatest stability. The utricle is found to be the most stress prone structure in the vestibular labyrinth and therefore appears to be the chamber most vulnerable to distention and potential modification.

## 1. Introduction

**Figure 1:**
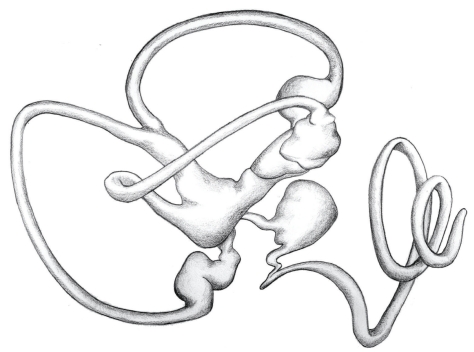
Artist's depiction of a reconstructed human labyrinth.

The membranous labyrinth is thought to have evolved from a simple tubular structure in primitive fish to its complex configuration in mammals [1]. The phylogeny of the process involves the sequential addition of structures. The simplest structure of the octavolateralis system is the tubular lateral line organ that arose in early fish [2]. Progressively more elaborate are (1) the primitive hagfish with a single canal with two ampullae joining the utricle, (2) the river lamprey with two separate canals each with its own ampulla joining the utricle, and (3) the Oyster toadfish with three ampullated canals in communication with the utricle as well as a saccule [3, 4]. Additional structures of lagaena and cochlea eventually evolved giving the mammalian labyrinth its current configuration. It is this sequential development that appears to have given the membranous labyrinth its cobbled together appearance as shown in Figure 1. 

While endophthalmic pressure plays an important role in the normal development of the eye [5], the role of endolymphatic pressure in the development of the ear's membranous labyrinth is unknown. The eye, because of its essentially spherical shape, enjoys a relatively uniform stress because of its symmetry [6]. The labyrinth, however, as its name implies, is substantially more complex in its configuration. The question then arises as to whether nature modulates the various physical attributes of the membranes to keep pressure-induced stress uniform throughout the labyrinthine membranes or, in contrast, tolerates significant disparities. Regional disparities in vulnerability to pressure would have the potential to effect asymmetric development. Membranous areas subject to greater stress would be more susceptible to distention and deformation. Individual differences, as emphasized by Darwin, could further accentuate any stress disparities and thus play a role in natural selection and evolution of the labyrinth [7]. 

**Figure 2:**
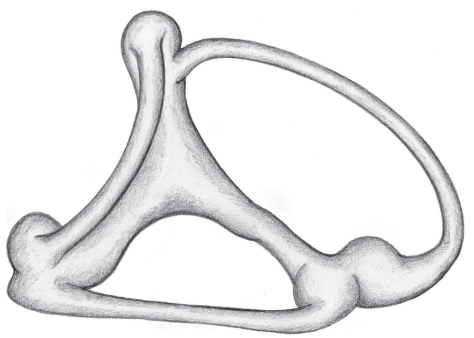
Artist's depiction of a reconstructed vestibular labyrinth in the Oyster Toadfish (after Ghanem).

Stress disparity can be studied using a previously reported analytical model [6]. The model has demonstrated that stress in the labyrinthine membranes is not necessarily uniform, but may vary from point to point depending on membrane thickness, degree of curvature, and chamber shape. In the current study, the analytical model will be used to analyze membrane hoop stress in the Oyster toadfish, a species widely used in inner ear research because of its large, readily exposed membranous labyrinth [4]. A reconstruction of the Oyster toadfish vestibular labyrinth is shown in Figure 2.

## 2. Methods and Materials

**Figure 3:**
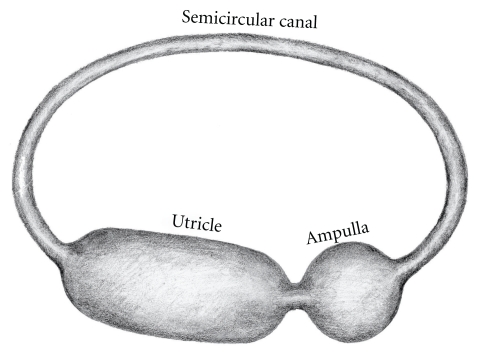
Model of the basic vestibular membranes (after Pender).

The model of the vestibular labyrinth consists of several membranous chambers joined in series, as shown in Figure 3. 

Here the semicircular canal is modeled as a thin toroid, the ampulla as a sphere, and the utricle as a thick cylinder. All membranes in the model are presumed subject to the same internal pressure because of the continuity of the internal volume. All membranes are presumed to be thin, that is, with no bending resistance [8]. (Thinness of a membrane is reflected in the ratio of radius to wall thickness (*r*/*w*). A value greater than five indicates that the diameter of the chamber is tenfold that of the wall thickness and constitutes an engineering criteria for thin [9]).

 Tensile stress in the toadfish labyrinth can be estimated using the analytic expressions developed for the vestibular model [6]. Using ellipsoidal shapes to emulate the various membranous chambers, the model analysis identified a geometric stress factor (GSF) that modulates transmural pressure in the production of hoop stress. This factor incorporates membrane thickness “*w*”, chamber radius “*r*”, as well as a shape parameter “*s*” reflecting the membranes configuration as indicated in

(1) $$\text{GSF}=\frac{\left(s\right)\left(r\right)}{w}.$$

In general, the shape determinant for peak hoop stress at the equator of an ellipsoid is given by

(2) $$\left(s\right)=\left(1-\frac{a^{2}}{2b^{2}}\right),$$

where “*a*” and “*b*” are the semiminor and semimajor dimensions of the ellipsoid. It should be noted that when “*b*” is infinite, the ellipsoid assumes a cylindrical form and the shape parameter becomes 1.0. When “*b*” is equal to “*a*”, the ellipsoid becomes spherical and the shape parameter “*s*” becomes 0.5.

The importance of the geometric stress factor lies in the fact that it modulates the effect of transmural pressure (*p*) in producing hoop stress (*t*) in the membranes as shown in

(3) $$t_{\text{hoop}}=\left(\text{GSF}\right)\left(p\right).$$

Thus both the transmural pressure and the geometric stress factor together determine the membrane peak membrane hoop stress at the equator of an ellipsoid. 

As to transmural pressure, a positive value, however slight [10], is assumed in the resting state to be just sufficient to keep the labyrinth inflated, and balanced by the intramural tensile stress [11]. Without a baseline positive pressure, the labyrinth's convex outer surface would be subject to collapse [12]. Such a positive pressure is presumably generated by active dark cell secretion of endolymph [13, 14]. Superimposed on the baseline static pressure are the inertial pressure oscillations resulting from head movements and stapes pulsations [15]. The latter appear to occur within the elastic limit of the vestibular membranes [16]. 

As to GSF, its value is dependent on the dimensional values of chamber shape proportions, membrane thickness, and radius of curvature, all of which must be measured. Optimal measurements would be made in situ and in vivo. Unfortunately, the tissues involved are microscopic and are not yet within the resolving power of current imaging techniques [17]. However, direct measurements made during surgery offer a reasonable alternative. (Another possible alternative source of data would entail measurements made from embedded tissue, applying a correction factor to overcome the inherent distortion due to fixation-contraction [18]. This methodology has been described elsewhere [4]. While less precise, this latter approach would permit assessment of archival tissues).

The published surgical data utilized in this study to analyze stress in the Oyster toadfish labyrinth are presented in Table 1. These measurements, taken from a population of normal fish, were reported as made during live animal surgery undertaken specifically to acquire such dimensional data [4]. Mean dimensions, along with maxima and minima, are shown. These reported data, while limited, are sufficiently detailed for the anterior canal, the lateral ampulla and the utricle to permit calculation of the geometric stress factors called for in the model.

Once the geometric stress factors have been determined for the various model chambers, normalized hoop stress can then be calculated. Normalized hoop stress (*t*_*n*_) is a comparative measure of the hoop stress, expressing the hoop stress in a particular chamber of the model relative to that in a reference chamber, as seen in (4). Inspection of (3) reveals that the hoop stress ratio between two chambers reduces to a ratio of its geometric stress factors, based on the assumption that all intralabyrinthine structures are exposed to the same transmural pressure:

(4) $$t_{n}=\left(\frac{t_{\text{hoop}1}}{t_{\text{hoop}2}}\right)=\;\frac{{\text{GSF}}_{1}}{{\text{GSF}}_{2}}.$$

Because of its comparative nature, normalized stress is a dimensionless number. Seen in this way, *t*_*n*_ is independent of pressure, and it is a convenient shorthand for summarizing the composite effect of structural elements on membrane stress. It does suggest that whatever the static transmural pressure, however slight, the reactive tensile hoop stress will be substantially higher in the chambers with the higher normalized stress values. Conversely, a low normalized stress level implies that a particular chamber is less vulnerable to the effects of transmural pressure and thus potentially more stable. In general, the chamber with the least geometric stress factor will be chosen as the reference chamber, thus implying that the normalized stress values will all have values equal to or greater than one.

## 3. Results

Results for the Oyster toadfish model analysis are presented in Table 2. 

The value of the membrane shape coefficients, 1.0 for a cylinder and 0.5 for a sphere, are derived from (2) as noted in Table 2. The membrane thinness ratios, based on the tissue dimensions, indicate that all values approach or exceed the thin membrane criteria of 5 or more, implying that a thin membrane analysis can be applied to the toadfish labyrinth. The geometric stress factor for each model compartment was computed from the membrane shape coefficients and membrane ratio values using (1). These values indicate that the anterior semicircular canal has the lowest mean geometric stress factor (4.4), reflecting its low radius of curvature and relatively thick membranes and its uniclastic cylindrical shape. The lateral ampulla's mean geometric stress factor (6.0) is higher than the canal's despite its advantageous spherical synclastic configuration mainly due to its thinner membranes and greater diameter. That of the utricle (17.4) is higher still, mainly due to its even thinner membrane and its suboptimal cylindrical shape. 

**Figure 4:**
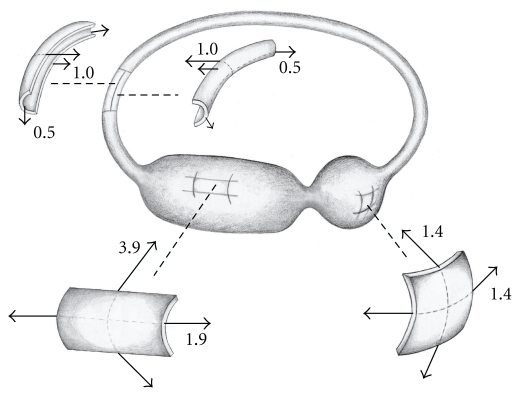
Normalized hoop stress in the oyster toadfish model. The breakout sections reflect the configuration of the membrane elements and indicate the attendant mean normalized stress levels in the different chambers.

Normalized values of mean hoop stress for the model toadfish labyrinth were then computed. Since the calculations indicate that the geometric stress factor in the semicircular canal represents the minimum value, normalized stress computations were based on this structure. Therefore, by definition, the mean value of the normalized hoop stress in the semicircular canal of the model is one (1.0). Normalized mean hoop stress level in the lateral ampulla (1.4) is less than double that in the canal while mean hoop stress in the utricle (3.9) is almost quadruple that in the canal. These results are interpreted graphically in the breakout membrane elements in Figure 4. 

Here the hoop stress vector is shown largest in the broad cylindrical utricle, less in the spherical ampulla, and least in the narrow semicircular canal. As previously reported and illustrated in Figure 4, axial stresses in the cylindrical sections of utricle and semicircular canal are one half those of their respective hoop stresses, while in the spherical ampullary section hoop stress is constant in all directions [6]. 

**Figure 5:**
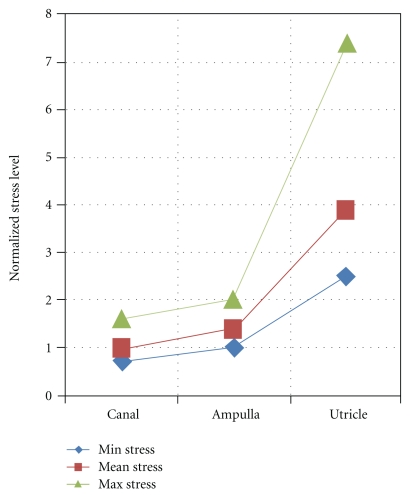
Normalized stress levels in the toadfish vestibular membranes. Mean, maximum, and minimum individual values for each of the three vestibular chambers are shown graphically.

When individual differences are considered, the computed interchamber stress disparities are even greater. This is displayed in Figure 5. 

This shows that the individual canal values are tightly clustered around a normalized stress value of 1.0 (with those of the ampulla fractionally higher) while the degree of splay in the utricle values is much more pronounced, with one individual displaying a normalized stress value 8-fold the canal minimum.

## 4. Discussion

In this analysis, it is seen that the distribution of stress is not uniform in the toadfish labyrinth. Mean stress levels are lowest for the semicircular canal, somewhat higher for the ampulla, and substantially higher for the utricle. This is the result of the composite influence of membrane shape, size, and thickness on membrane stress. In theory, the interplay between the various stress determinants of membrane shape size and thickness could result, on the one hand, in situations where one determinant counterbalances another to keep stress moderate. On the other hand, one determinant could exaggerate the effects of another to propel stress to higher levels. In the end, it is the composite effect of the three factors working in unison that is important. This composite effect can be examined chamber by chamber to better understand this phenomenon.

The toadfish anterior semicircular canal is basically a narrow tube with a slight bend. Its higher suboptimal uniclastic shape (1.0) is offset by its narrow bore (307 *μ*) and thicker membranes (70 *μ*). This composite effect gives the semicircular canal the lowest computed mean geometric stress factor (4.4) and lowest normalized stress of the three chambers. Even accounting for its actual toroidal shape with its anticlastic inner surface would increase the stress only by 5% [6]. These facts suggest that the semicircular canal from a membrane stability point of view would be predicted to be the structure least vulnerable to stress and consequently the most stable of the three chambers analyzed in the toadfish vestibular labyrinth. This may well reflect the evolutionary age of this ancient structure, with its probable origin in the lateral line organ of early fish prior to the Ordovician period and dating back more than 400 million years [2].

The ampulla appears to be a dilated section of the semicircular canal that has a spheroidal shape. Its increased radial size (697 *μ*) tends to increase its stress levels compared to that of the adjoining semicircular canal (307 *μ*). Overall its membranes are thinner (58 *μ*) than those of the semicircular canal (70 *μ*), further aggravating mean stress. However, these two adverse effects on stress are largely compensated by its favorable spherical shape determinant (0.5) that ameliorates stress. This composite effect gives the ampulla only a fractionally higher geometric stress factor (6.0) than that estimated for the semicircular canal (4.4). This fact underscores the importance of the advantageous spherical shape in lessening stress [19]. In fact, the overlap in individual stress values of the ampulla with the semicircular canal as shown in Figure 5 demonstrates that in at least some instances, a spherical ampulla can be associated with lower stresses than found in some semicircular canals. Within certain geometric limits, transition from a tubular to a spherical shape can be associated with a net reduction in hoop stress and thus represent a safety valve should an area of the tubular canal structure weaken. This illustrates the concept that spherical dilation can act to moderate stress in a tubular structure and thus promote membrane stability. This would be particularly important in the case of pressure surges in a closed semicircular canal system. This raises the possibility that the ampullary structure may represent a canal dilation in response to pressure. 

The utricle is a basically a shared tubular chamber that connects the several canals. It has an increased radial size (556 *μ*) at its narrowest point compared to the semicircular canal (307 *μ*), and dilated regions will experience greater stress. It is modeled as a cylinder with a conservative uniclastic shape (determinant 1.0). (Hourglass areas that have an anticlastic shape will have a shape determinant greater than 1.0 entailing even higher stress levels). The remaining determinant, membrane thickness, could theoretically offset the above two stressful tendencies but such is observed not to be the case. Instead, the utricular membrane (32 *μ*) is found to be the thinnest of the three chambers. As a consequence the composite effect of the three stress determinants is to propel mean utricular membrane stress (17.4) to quadruple the levels experienced by the semicircular canal. And to compound matters further, individual differences in utricular membrane thickness can result in stress levels double the mean resulting in stress levels 8× the canal minimum. This tends to make the utricle the most stress prone chamber in the vestibular labyrinth. Such an enhancement of utricular stress by individual differences would be accompanied by increased probability of structural instability in the face of pressure excursions. This would make the utricle a prime candidate for stress-induced distention and a potential nidus for evolutionary modification. This underscores the importance of individual differences in driving evolution as emphasized by Darwin [7].

The findings described herein are pertinent to the question raised previously [6] as to whether natural adjustments in the individual parametric determinants of stress in the various chambers occur to keep peak hoop stress more or less a constant value throughout the membranous labyrinth. The stress disparities reported here indicate that this is not the case, especially for the utricle whose estimated stress levels are substantially above those in the semicircular canal. 

 This lack of parity further fuels speculation that such structures themselves may represent progenitor forms, one giving rise to the next in a serial manner, resulting in the cobbled together appearance of the membranous labyrinth, particularly in mammals as seen in Figure 1. The spherical ampulla may have evolved from the primitive semicircular canal as a dilation in response to pressure. A more ovoid ampulla would represent a further metamorphosis from the perfectly spherical, and the cylindroidal utricle an even greater one. Since these shapes are part of a configurational ellipsoidal continuum [6], the exact shape that a particular chamber assumes may be in fact dependent on the magnitude of the driving transmural pressure. The shape adjusting mechanism would likely involve loss of membrane mechanical advantage as the membrane distends in the area affected and may be limited only by increasing material stiffness as distention proceeds.

## 5. Conclusions

The model predicts that substantial hoop stress disparities exist in the toadfish vestibular labyrinth. The minimum stress level computed for the semicircular canal suggests that this chamber is the least vulnerable to transmural pressure. Fractionally higher stress levels encountered in the ampulla suggest a somewhat greater vulnerability. However, substantially higher mean stress levels are projected for the utricle in its normal baseline configuration. Additionally, the effect of individual differences in membrane configuration exaggerates these disparities even further. Stress disparities in some individual toadfish may rise to and possibly exceed an order of magnitude. Such disparities in stress susceptibility may be a measure of differences in membrane stability as well as reflecting a vulnerability to pressure-mediated metamorphosis. If such be the case, then the model predicts that the toadfish semicircular canal is the most stable structure in the basic membranous labyrinth while the utricle appears to be the least stable and most vulnerable to pressure driven distention. This may have imbued the membrane structure of the utricle with a greater potential to undergo evolutive modification in service of its contained sensory epithelium.

##  Conflict of Interest

The author declares that he has no conflict of interests.

## Figures and Tables

**Table 1:** Live measurements of the toadfish labyrinth*.

Membrane structure	Mean thickness “*w*” (max/min) in microns	Mean radius of curvature “*r*” (max/min) in microns
Anterior Canal	70 (86/45)	307 (322/270)
Lateral Ampulla	58 (75/44)	697 (788/625)
Utricle	32 (42/20)	556 (654/469)

*Measurements were made during surgery in live fish reported elsewhere [4]. Mean values reflect 12–25 measurements from 3–5 fish along with maximal and minimal individual values. Utricle was measured at its narrowest point.

**Table 2:** Mean membrane parameters for the toadfish labyrinth*.

Membrane structure	Membrane model shape	Membrane shape coefficient “*s*”	Membrane thinness ratio “*r*/*w*”	Membrane geometric stress factor “(*s*)(*r*/*w*)”	Membrane hoop stress normalized^#^ “*t*_*n*_”
Ant. Canal	Cylinder	1.0	4.4	4.4	1.0
Lat. Ampulla	Sphere	0.5	12.0	6.0	1.4
Utricle	Cylinder	1.0	17.4	17.4	3.9

*Based on mean dimensional measurements [4].

^#^Based on the semicircular canal as reference.
